# Methyl 3-[(*E*,*E*)-3-phenyl­prop-2-enyl­idene]dithio­carbazate

**DOI:** 10.1107/S1600536810041115

**Published:** 2010-10-20

**Authors:** M. T. H. Tarafder, Sultana Shakila Khan, M. A. A. A. A. Islam, Lea Lorenzi, Ennio Zangrando

**Affiliations:** a Department of Chemistry, Rajshahi University, Rajshahi 6205, Bangladesh; b Department of Chemistry, Rajshahi University of Engineering & Technology, Rajshahi 6204, Bangladesh; c Dipartimento di Scienze Chimiche, Via Licio Giorgieri 1, 34127 Trieste, Italy

## Abstract

In the title compound, C_11_H_12_N_2_S_2_, the dithio­carbazate group adopts an *EE* configuration with respect to the C=C and C=N bonds of the propenyl­idene group. The atoms of the propenyl­idene and dithio­carbazate unit are essentially co-planar, with a maximum deviation of 0.058 (1) Å; the phenyl ring forms a dihedral angle of 18.3 (1)° with this fragment. In the crystal, mol­ecules form inversion dimers *via*  pairs of N—H⋯S hydrogen bonds involving the terminal S atom.

## Related literature

For the synthesis and a related structure, see: Tarafder *et al.* (2008 ▶). 
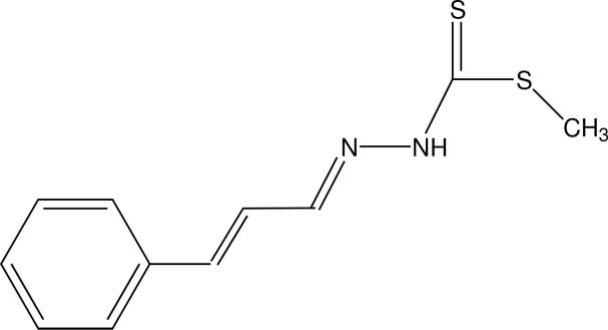


## Experimental

### 

#### Crystal data

C_11_H_12_N_2_S_2_
*M* *_r_* = 236.35
Monoclinic, 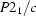

*a* = 10.408 (2) Å
*b* = 5.4950 (9) Å
*c* = 20.988 (2) Å
β = 100.697 (10)°
*V* = 1179.5 (3) Å^3^
*Z* = 4
Mo *K*α radiation
μ = 0.42 mm^−1^
*T* = 293 K
0.40 × 0.15 × 0.12 mm

#### Data collection

Enraf–Nonius DIP1030 image-plate diffractometer
6316 measured reflections
2048 independent reflections
1569 reflections with *I* > 2.0σ(*I*)
*R*_int_ = 0.037

#### Refinement

*R*[*F* ^2^ > 2σ(*F* ^2^)] = 0.038
*wR*(*F* ^2^) = 0.116
*S* = 1.04
2048 reflections
138 parameters
H-atom parameters constrained
Δρ_max_ = 0.14 e Å^−3^
Δρ_min_ = −0.21 e Å^−3^

### 

Data collection: *XPRESS* (MacScience, 2002 ▶); cell refinement: *DENZO* (Otwinowski & Minor, 1997 ▶); data reduction: *DENZO* and *SCALEPACK* (Otwinowski & Minor, 1997 ▶); program(s) used to solve structure: *SHELXS97* (Sheldrick, 2008 ▶); program(s) used to refine structure: *SHELXL97* (Sheldrick, 2008 ▶); molecular graphics: *ORTEP-3 for Windows* (Farrugia, 1997 ▶); software used to prepare material for publication: *WinGX* (Farrugia, 1999 ▶).

## Supplementary Material

Crystal structure: contains datablocks I, global. DOI: 10.1107/S1600536810041115/pv2336sup1.cif

Structure factors: contains datablocks I. DOI: 10.1107/S1600536810041115/pv2336Isup2.hkl

Additional supplementary materials:  crystallographic information; 3D view; checkCIF report

## Figures and Tables

**Table 1:** Hydrogen-bond geometry (Å, °)

*D*—H⋯*A*	*D*—H	H⋯*A*	*D*⋯*A*	*D*—H⋯*A*
N1—H1⋯S1^i^	0.86	2.67	3.4086 (19)	145

Symmetry code: (i) 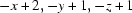.

**Fig. 1.**
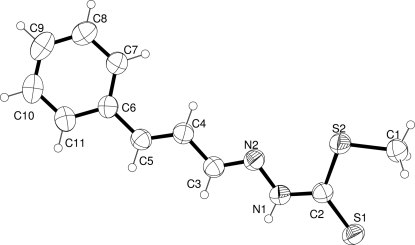
ORTEP drawing of the title molecule; thermal ellipsoids are drawn at the 40% probability level.

## supplementary crystallographic information

### Comment

The molecule of the title compound is shown in Fig. 1. The atoms of propenylidene and dithiocarbazate moiety lie essentially in the same plane, with a maximum deviation from the mean plane of 0.058 (1) Å exhibited by S2, while the phenyl ring forms a dihedral angle of 18.3 (1)° with the cited fragment. The crystal packing evidences molecules connected in pairs about inversion centers and connected by N1—H1···S1 hydrogen bonds involving the terminal sulfur atom (Table 1). The structural features of the title compound are smilar to those observed in a benzyl derivative (Tarafder *et al.*, 2008)). The Schiff base is potentially bidentate and coordinates *via* the β-nitrogen and the thiolate anion generated during complexation.

### Experimental

The *S*-methyldithiocarbazate (2.44 g, 0.2 mol), prepared as previously described (Tarafder *et al.*, 2008), was dissolved in hot absolute ethanol (30–40 ml). To this solution an equimolar amount of cinnamaldehyde in hot absolute ethanol (20 ml) was added and the mixture was heated for 20 min and then cooled. The orange precipitate thus formed was separated and dried *in vacuo* over anhydrous CaCl_2_. Orange needle shaped single crystals of the Schiff base were obtained after recrysallization from acetone over 15 days; *M*. p. 443 K (very sharp and abrupt).

### Refinement

All H atoms were located geometrically and treated as riding atoms, with N—H = 0.86 and C—H = 0.93 and 0.96 Å for aryl and methyl H-atoms with *U*ĩso~(H) = 1.2*U*~eq~(N or aryl-C) or 1.5*U*~eq~(methyl-C).

### Crystal data

**Table:**

C_11_H_12_N_2_S_2_	*F*(000) = 496
*M**_r_* = 236.35	*D*_x_ = 1.331 Mg m^−^^3^
Monoclinic, *P*2_1_/*c*	Mo *K*α radiation, λ = 0.71073 Å
Hall symbol: -P 2ybc	Cell parameters from 275 reflections
*a* = 10.408 (2) Å	θ = 3.6–20.7°
*b* = 5.4950 (9) Å	µ = 0.42 mm^−^^1^
*c* = 20.988 (2) Å	*T* = 293 K
β = 100.697 (10)°	Needle, orange
*V* = 1179.5 (3) Å^3^	0.40 × 0.15 × 0.12 mm
*Z* = 4	

### Data collection

**Table:**

Enraf–Nonius DIP1030 image-plate diffractometer	1569 reflections with *I* > 2.0σ(*I*)
Radiation source: fine-focus sealed tube	*R*_int_ = 0.037
graphite	θ_max_ = 25.3°, θ_min_ = 3.1°
φ–scans with narrow frames	*h* = −12→12
6316 measured reflections	*k* = −6→6
2048 independent reflections	*l* = −25→25

### Refinement

**Table:**

Refinement on *F*^2^	Secondary atom site location: difference Fourier map
Least-squares matrix: full	Hydrogen site location: inferred from neighbouring sites
*R*[*F*^2^ > 2σ(*F*^2^)] = 0.038	H-atom parameters constrained
*wR*(*F*^2^) = 0.116	*w* = 1/[σ^2^(*F*_o_^2^) + (0.0721*P*)^2^] where *P* = (*F*_o_^2^ + 2*F*_c_^2^)/3
*S* = 1.04	(Δ/σ)_max_ = 0.001
2048 reflections	Δρ_max_ = 0.14 e Å^−^^3^
138 parameters	Δρ_min_ = −0.21 e Å^−^^3^
0 restraints	Extinction correction: *SHELXL*, Fc^*^=kFc[1+0.001xFc^2^λ^3^/sin(2θ)]^-1/4^
Primary atom site location: structure-invariant direct methods	Extinction coefficient: 0.023 (4)

### Special details

**Table:**

Geometry. All s.u.'s (except the s.u. in the dihedral angle between two l.s. planes) are estimated using the full covariance matrix. The cell s.u.'s are taken into account individually in the estimation of s.u.'s in distances, angles and torsion angles; correlations between s.u.'s in cell parameters are only used when they are defined by crystal symmetry. An approximate (isotropic) treatment of cell s.u.'s is used for estimating s.u.'s involving l.s. planes.
Refinement. Refinement of *F*^2^ against ALL reflections. The weighted *R*-factor *wR* and goodness of fit *S* are based on *F*^2^, conventional *R*-factors *R* are based on *F*, with *F* set to zero for negative *F*^2^. The threshold expression of *F*^2^ > 2σ(*F*^2^) is used only for calculating *R*-factors(gt) *etc*. and is not relevant to the choice of reflections for refinement. *R*-factors based on *F*^2^ are statistically about twice as large as those based on *F*, and *R*- factors based on ALL data will be even larger.

### Fractional atomic coordinates and isotropic or equivalent isotropic displacement parameters (Å^2^)

**Table:**

	*x*	*y*	*z*	*U*_iso_*/*U*_eq_	
S1	1.15322 (6)	0.68232 (10)	0.57196 (3)	0.0759 (2)	
S2	1.08034 (6)	0.51686 (10)	0.69852 (3)	0.0734 (2)	
N1	0.99960 (18)	0.3187 (3)	0.58644 (8)	0.0682 (5)	
H1	0.9962	0.2915	0.5458	0.082*	
N2	0.92717 (19)	0.1751 (3)	0.62061 (9)	0.0680 (5)	
C1	1.1813 (3)	0.7789 (4)	0.72034 (11)	0.0791 (6)	
H1A	1.2653	0.7525	0.7089	0.119*	
H1B	1.1918	0.8059	0.7662	0.119*	
H1C	1.1407	0.9187	0.6976	0.119*	
C2	1.0751 (2)	0.4996 (3)	0.61523 (10)	0.0625 (5)	
C3	0.8633 (2)	0.0018 (3)	0.58766 (11)	0.0656 (5)	
H3	0.8723	−0.0225	0.5449	0.079*	
C4	0.7788 (2)	−0.1538 (3)	0.61562 (10)	0.0670 (5)	
H4	0.7718	−0.1294	0.6587	0.080*	
C5	0.7095 (2)	−0.3328 (3)	0.58259 (10)	0.0653 (5)	
H5	0.7240	−0.3613	0.5408	0.078*	
C6	0.6139 (2)	−0.4880 (3)	0.60503 (10)	0.0634 (5)	
C7	0.5705 (3)	−0.4487 (4)	0.66304 (12)	0.0750 (6)	
H7	0.6053	−0.3208	0.6898	0.090*	
C8	0.4770 (3)	−0.5962 (5)	0.68147 (14)	0.0898 (7)	
H8	0.4487	−0.5659	0.7202	0.108*	
C9	0.4250 (3)	−0.7874 (5)	0.64316 (17)	0.0963 (9)	
H9	0.3623	−0.8870	0.6560	0.116*	
C10	0.4657 (3)	−0.8309 (4)	0.58615 (16)	0.0944 (8)	
H10	0.4305	−0.9603	0.5601	0.113*	
C11	0.5596 (3)	−0.6831 (4)	0.56675 (13)	0.0799 (7)	
H11	0.5866	−0.7146	0.5277	0.096*	

### Atomic displacement parameters (Å^2^)

**Table:**

	*U*^11^	*U*^22^	*U*^33^	*U*^12^	*U*^13^	*U*^23^
S1	0.0761 (5)	0.0872 (4)	0.0662 (4)	−0.0152 (3)	0.0178 (3)	0.0061 (2)
S2	0.0809 (5)	0.0799 (4)	0.0592 (4)	−0.0143 (3)	0.0122 (3)	0.0035 (2)
N1	0.0743 (13)	0.0726 (10)	0.0600 (10)	−0.0089 (9)	0.0181 (9)	−0.0002 (7)
N2	0.0681 (12)	0.0698 (10)	0.0670 (10)	−0.0066 (8)	0.0150 (9)	0.0030 (8)
C1	0.0811 (17)	0.0813 (13)	0.0730 (14)	−0.0110 (12)	0.0092 (12)	−0.0046 (10)
C2	0.0574 (13)	0.0664 (12)	0.0636 (12)	0.0012 (9)	0.0112 (9)	0.0024 (8)
C3	0.0686 (15)	0.0647 (12)	0.0631 (12)	0.0011 (10)	0.0111 (10)	0.0014 (8)
C4	0.0698 (15)	0.0673 (11)	0.0631 (12)	−0.0028 (10)	0.0104 (10)	0.0010 (9)
C5	0.0682 (14)	0.0658 (12)	0.0611 (11)	0.0013 (10)	0.0099 (10)	−0.0002 (8)
C6	0.0633 (14)	0.0560 (10)	0.0683 (13)	0.0023 (8)	0.0057 (10)	0.0036 (8)
C7	0.0799 (17)	0.0700 (12)	0.0756 (14)	−0.0059 (11)	0.0160 (12)	0.0006 (10)
C8	0.090 (2)	0.0870 (15)	0.0964 (18)	−0.0020 (14)	0.0276 (15)	0.0160 (13)
C9	0.0772 (18)	0.0813 (16)	0.129 (3)	−0.0085 (13)	0.0151 (17)	0.0274 (16)
C10	0.091 (2)	0.0722 (15)	0.114 (2)	−0.0154 (13)	0.0022 (17)	−0.0017 (13)
C11	0.0866 (18)	0.0680 (13)	0.0813 (15)	−0.0038 (12)	0.0061 (13)	−0.0054 (10)

### Geometric parameters (Å, °)

**Table:**

S1—C2	1.664 (2)	C5—C6	1.454 (3)
S2—C2	1.741 (2)	C5—H5	0.9300
S2—C1	1.791 (2)	C6—C7	1.392 (3)
N1—C2	1.339 (3)	C6—C11	1.395 (3)
N1—N2	1.381 (2)	C7—C8	1.376 (3)
N1—H1	0.8600	C7—H7	0.9300
N2—C3	1.287 (3)	C8—C9	1.372 (4)
C1—H1A	0.9600	C8—H8	0.9300
C1—H1B	0.9600	C9—C10	1.363 (4)
C1—H1C	0.9600	C9—H9	0.9300
C3—C4	1.428 (3)	C10—C11	1.388 (4)
C3—H3	0.9300	C10—H10	0.9300
C4—C5	1.335 (3)	C11—H11	0.9300
C4—H4	0.9300		
			
C2—S2—C1	102.02 (11)	C4—C5—H5	116.6
C2—N1—N2	121.39 (18)	C6—C5—H5	116.6
C2—N1—H1	119.3	C7—C6—C11	117.4 (2)
N2—N1—H1	119.3	C7—C6—C5	123.05 (18)
C3—N2—N1	114.92 (18)	C11—C6—C5	119.5 (2)
S2—C1—H1A	109.5	C8—C7—C6	121.1 (2)
S2—C1—H1B	109.5	C8—C7—H7	119.5
H1A—C1—H1B	109.5	C6—C7—H7	119.5
S2—C1—H1C	109.5	C9—C8—C7	120.6 (3)
H1A—C1—H1C	109.5	C9—C8—H8	119.7
H1B—C1—H1C	109.5	C7—C8—H8	119.7
N1—C2—S1	120.44 (16)	C10—C9—C8	119.7 (3)
N1—C2—S2	113.58 (15)	C10—C9—H9	120.1
S1—C2—S2	125.98 (12)	C8—C9—H9	120.1
N2—C3—C4	121.2 (2)	C9—C10—C11	120.4 (2)
N2—C3—H3	119.4	C9—C10—H10	119.8
C4—C3—H3	119.4	C11—C10—H10	119.8
C5—C4—C3	122.8 (2)	C10—C11—C6	120.8 (3)
C5—C4—H4	118.6	C10—C11—H11	119.6
C3—C4—H4	118.6	C6—C11—H11	119.6
C4—C5—C6	126.9 (2)		
			
C2—N1—N2—C3	−177.27 (18)	C4—C5—C6—C11	−173.6 (2)
N2—N1—C2—S1	−175.70 (15)	C11—C6—C7—C8	−0.5 (3)
N2—N1—C2—S2	4.4 (2)	C5—C6—C7—C8	178.1 (2)
C1—S2—C2—N1	−177.67 (16)	C6—C7—C8—C9	0.6 (4)
C1—S2—C2—S1	2.41 (18)	C7—C8—C9—C10	−0.4 (4)
N1—N2—C3—C4	−176.76 (18)	C8—C9—C10—C11	0.1 (4)
N2—C3—C4—C5	178.9 (2)	C9—C10—C11—C6	0.0 (4)
C3—C4—C5—C6	−174.93 (19)	C7—C6—C11—C10	0.2 (3)
C4—C5—C6—C7	7.9 (3)	C5—C6—C11—C10	−178.4 (2)

### Hydrogen-bond geometry (Å, °)

**Table:**

*D*—H···*A*	*D*—H	H···*A*	*D*···*A*	*D*—H···*A*
N1—H1···S1^i^	0.86	2.67	3.4086 (19)	145

Symmetry codes: (i) −*x*+2, −*y*+1, −*z*+1.
